# Food safety concerns deriving from the use of silver based food packaging materials

**DOI:** 10.3389/fmicb.2015.01109

**Published:** 2015-10-09

**Authors:** Alessandra Pezzuto, Carmen Losasso, Marzia Mancin, Federica Gallocchio, Alessia Piovesana, Giovanni Binato, Albino Gallina, Alberto Marangon, Renzo Mioni, Michela Favretti, Antonia Ricci

**Affiliations:** ^1^Optimization and Control of Food Production Laboratory, Istituto Zooprofilattico Sperimentale delle VenezieSan Donà di Piave, Italy; ^2^Department of Food Safety, Istituto Zooprofilattico Sperimentale delle VenezieLegnaro, Italy; ^3^Laboratory of Chemistry, Istituto Zooprofilattico Sperimentale delle VenezieLegnaro, Italy; ^4^Sensory Analysis Laboratory, Veneto Agricoltura, Istituto per la Qualità e le Tecnologie AgroalimentariThiene, Italy

**Keywords:** food safety, food packaging, antimicrobial, silver migration

## Abstract

The formulation of innovative packaging solutions, exerting a functional antimicrobial role in slowing down food spoilage, is expected to have a significant impact on the food industry, allowing both the maintenance of food safety criteria for longer periods and the reduction of food waste. Different materials are considered able to exert the required antimicrobial activity, among which are materials containing silver. However, challenges exist in the application of silver to food contact materials due to knowledge gaps in the production of ingredients, stability of delivery systems in food matrices and health risks caused by the same properties which also offer the benefits. Aims of the present study were to test the effectiveness and suitability of two packaging systems, one of which contained silver, for packaging and storing *Stracchino* cheese, a typical Italian fresh cheese, and to investigate if there was any potential for consumers to be exposed to silver, *via* migration from the packaging to the cheese. Results did not show any significant difference in the effectiveness of the packaging systems on packaged *Stracchino* cheese, excluding that the active packaging systems exerted an inhibitory effect on the growth of spoilage microorganisms. Moreover, silver migrated into the cheese matrix throughout the storage time (24 days). Silver levels in cheese finally exceeded the maximum established level for the migration of a non-authorised substance through a functional barrier (Commission of the European Communities, 2009). This result poses safety concerns and strongly suggests the need for more research aimed at better characterizing the new packaging materials in terms of their potential impacts on human health and the environment.

## Introduction

Food packaging is continuously evolving in response to growing consumer demand for minimally processed, more natural, fresh, and longer storable food.

Moreover, every year an increasing amount of edible food is lost along the entire food supply chain. The European Commission estimated that the annual food waste amounts to 89 million tons, or 179 kg per capita, varying considerably between individual countries and the various sectors, without even considering agricultural food waste or fish catches returned to the sea; furthermore, total food waste is expected to rise to approximately 126 million tons (a 40% increase) by 2020, unless additional preventive actions or measures are taken (European Parliament, 2012; European Parliament – STOA, 2013). Thus, packaging optimisation strategies have been proposed to guarantee the maintenance of food safety criteria and reduce food waste.

This scenario has strongly inspired the packaging industry to go beyond the traditional functions of the package and to offer innovative solutions addressing the changing demands of the food industry and consumers as well as the increasing regulatory and legal requirements (Realini and Marcos, 2014). Therefore, the function of food packaging has evolved from being a simple physical barrier, aimed at avoiding food contact with the external environment (passive packaging), to exerting a functional role in slowing down food spoilage (active packaging) by means of specific action on the chemical, enzymatic and mechanical phenomena, thus extending the shelf life of food (Marsh and Bugusu, 2007).

Different active packaging products are considered able to exert antimicrobial activity, especially when the materials contain silver (Quintavalla and Vicini, 2002; Coma, 2008). However, even though silver’s antibacterial properties have long been proved (Silver et al., 2006), the lack of standardization in terms of particle characterization and test conditions makes it difficult to define its range of effectiveness and specificity against different bacterial species (Bae et al., 2010).

Moreover, there is an expanding body of scientific studies demonstrating that silver, especially in its nanosize, could introduce new risks to human health (Ahamed et al., 2010; Gaillet and Rouanet, 2015). The use of silver in food contact materials could potentially increase the probability of consumers’ exposure: silver could migrate from packaging into foods, even though preliminary results indicate that migration is expected to be minimal (Chaudhry et al., 2008); although these studies seem to give some reassurances about safety, the few migration studies published to date have been targeted at food simulants, so further investigation needs to be performed especially in the case of complex food matrices.

In the European Union, the main regulatory framework related to the use of food contact materials is still Regulation (EC) No (1935/2004); it states that “*materials and articles, including active and intelligent materials and articles do not have to transfer their constituents to food in quantities which could endanger human health or bring about an unacceptable change in the composition of the food or bring about a deterioration in the organoleptic characteristics thereof.*” However, the scientific literature has a complete lack of any data quantifying rates of migration of food package components into food.

The European Food Safety Authority stipulated, with reference to article 10 of Regulation (EC) No (1935/2004), an opinion of the panel on food contact materials, enzymes, flavourings and processing aids (CEF) of the risks originating from the migration of substances from food contact materials into food is required [European Food Safety Authority (EFSA), 2009]. According to Commission of the European Communities (2009), Article 14, a maximum level of migration of 0.001 mg/kg should be observed for the migration of a non-authorized substance through a functional barrier. For some novel substances intended for inclusion in food packaging materials, such as silver, adequate toxicological data is not yet available and so safety assessments are still in progress. Thus, silver must undergo an appropriate authorisation process and safety evaluation before it is introduced.

Aims of the present study were to test the effectiveness of two active packaging systems, one of which contained silver, for the packaging and storage at 4°C of *Stracchino* cheese, a typical Italian fresh cheese, by assessing microbial, chemical, and sensorial parameters. Moreover, the migration of silver from the packaging into the cheese was monitored during the chill storage period.

## Materials and Methods

### Materials

Two active food packaging systems were studied in the present research: the active packaging *Food-touch*^®^ by Microbeguard Corp. (USA), containing silver zeolite for the purpose of exerting antimicrobial properties, and an innovative packaging, *Ovtene*^®^ by Arcadia Spa (Italy), containing calcium carbonate, talc and titanium dioxide, for the purpose of acting as an absorber with the intent to extend food shelf life and supplied by a local producer. Both of these products were used in their original liner formats, as provided by suppliers. As control, a traditional passive packaging system was also used.

*Stracchino* cheese, a commercially available typical Italian fresh cheese with a declared shelf life of 20 days, preferentially consumed by children and the elderly, was selected as a perishable and high value food suitable for use in these packaging systems.

*Stracchino* pieces (250 g) were hand-wrapped according to manufacturers’ instructions in the selected packaging systems and then analyzed for a range of microbial, chemical and sensorial parameters. Packaged *Stracchino* cheese was stored at 4°C for 25 days.

### Microbial, Water Activity, and pH Analyses

Microbial analyses were conducted to monitor the numbers of spoilage and indicator microorganisms in the cheese during storage in the three selected packaging systems as detailed in **Table 1**.

**Table 1:** Sampling design for microbial, chemical, and sensorial determination.

		Number of samples		
Experimental time (days)	Storage temperature (°C)	Traditional packaging	Innovative packaging (*Ovtene^®^*)	Active packaging (*Food-touch^®^*)
0	0/+4	20	–	–
8	0/+4	20	20	20
15	0/+4	20	20	20
21	0/+4	20	20	20
25	0/+4	20	20	20
Total number of samples		100	80	80

The following microbial determinations were performed:

Total Viable Count at 30°C (ISO 4833, 2003a; Plate Count Agar at 30±1°C for 72±3 h in aerobic conditions), *Pseudomonas* spp. (Cetrimide-Fusidic acid-Cephalosporin Agar at 25±1°C for 44±4 h), *Enterobacteriaceae* (ISO 21528-2, 2004a; Violet Red Bile Glucose Agar at 37±1°C for 24±2 h), Lactic Acid Bacteria at 30°C (MRS Agar at 30±1°C for 72±3 h in aerobic conditions), Moulds and Yeasts (Rose Bengal Chloramphenicol Agar at 25±1°C for 3–5 days). Additionally, water activity was measured (*a_w_*, according to ISO 21807, 2004b), as was pH (ISO 2917, 1999).

### Chemical Analyses

Chemical analyses were conducted on days 0, 7, 14, 20, and 24 of storage. In total, 20 replicate cheese samples were examined each day. The following chemical parameters were assessed:

(1) *Total Volatile Basic Nitrogen* (TVB-N), determined by the method prescribed in Commission of the European Communities (2005) for the evaluation of the TVN-B in fish;
(2) *Sulfides*, determined by Lead Acetate Test Strips (Sigma-Aldrich, Switzerland);
(3) *Peroxides* via iodometric titration (Biffoli, 1979);
(4) *Stamm test* (Hamm et al., 1965);
(5) *Thiobarbituric acid test* (TBA; Fernandez et al., 1997).

### Sensory Evaluation

In order to detect any sensory difference during the storage period, sensory evaluation was conducted on *Stracchino* cheese packaged using only the *Ovtene*^®^ system and the traditional packaging as control. Sensory evaluation on *Stracchino* packaged in the *Food-touch*^®^ system was not conducted, due to the claimed presence of silver and the possibility of its migration into the cheese matrix. Two sensory evaluations were performed after 0 and 7 days of storage using fresh *Stracchino* as a standard reference. The sensory evaluation was performed as described by ISO 13299 (2003b). A panel of 15 judges, experts in sensory evaluation of cheese, was previously trained on the quantitative evaluation of the following selected descriptors: (i) *Odor intensity*; (ii) *Aroma intensity*; (iii) *Saltiness*; (iv) *Acidity*; (v) *Bitterness*; (vi) *Homogeneity*; (vii) *Consistency*; (viii) *Adherence.*

### Silver Migration Test

Silver migration from the *Food-touch*^®^ system into the cheese was determined on days 0, 7, 14, 20, and 24 of storage. In total, two independent determinations of 20 cheese samples were examined each day.

#### Atomic Absorption Instrumentation

Silver concentrations were determined using Electrothermal Atomic Absorption Spectrometry (ETAAS) on an M6 mkII Atomic Absorption Spectrometer (Thermo Electron, Cambridge, UK) with D_2_ background correction, equipped with a GF95 Graphite Furnace atomiser.

#### Analytical Methods

Cheese samples (2 g) were homogenized, then 8 ml concentrated HNO_3_ and 2 ml H_2_O_2_ were added and the mixture digested in Teflon liners using a CEM (Mattews, NC, USA) Mars Xpress microwave oven. Digested cheese samples were then diluted to up to 25 ml in class A volumetric flasks with deionised water and analyzed. All calibration solutions for metal determination were made by dilution from Certified Standard Solutions (ULTRAgrade^®^ ICP Standards, 1000 mg/mL) provided by Ultra Scientific (North Kingstown, RI, USA).

Trueness of analytical data was verified by means of Certified Reference Materials (NRCC DORM2) analyzed concurrently with samples in each analytical batch. LOQ (6 s) value were found to be 0.0015 mg/kg. Operating conditions are reported in Supplementary Table S1.

### Statistical Analysis

The microbial counts were logarithm transformed, and data distributions over time were represented by box and whiskers plots. To evaluate the effect of type of packaging, time and the interaction between the two independent variables on the dynamics of the microbial parameters, analysis of variance (ANOVA) was applied. The observation taken at point 0 (day 1), presenting the same values distribution for each type of packaging and the outliers, identified using Grubbs test, were not considered in the analysis (Grubbs, 1969; Agresti, 1990; Dohoo et al., 2003).

The assumption of homoscedasticity was verified using the Breusch-Pagan & Cook-Weisberg test and the residual plot (Breusch and Pagan, 1979; Cook and Weisberg, 1983).

The normality condition of residuals was verified using the Shapiro-Francia test (Shapiro and Francia, 1972), the graphical analysis of the residuals plotted against the normal probability distribution (q-q plot), and the histogram of residuals versus normal curve (Dohoo et al., 2003).

A post-estimation analysis was performed to evaluate the significant paired contrasts taking into account the multi test correction in the evaluation of statistical significance (Dohoo et al., 2003).

The software STATA 12.0 (Release 12) was used to conduct the statistical analysis of microbial data.

Analysis of variance was also performed to analyze the sensory data by SYSTAT software, in order to assess any significant sensory difference between cheese treated with the active or the traditional packaging systems, after checking the reliability and homogeneity of the obtained data.

*P*-value < 0.05 was considered significant in the statistical analysis.

## Results

### Microbial Analyses

**FIGURE 1:**
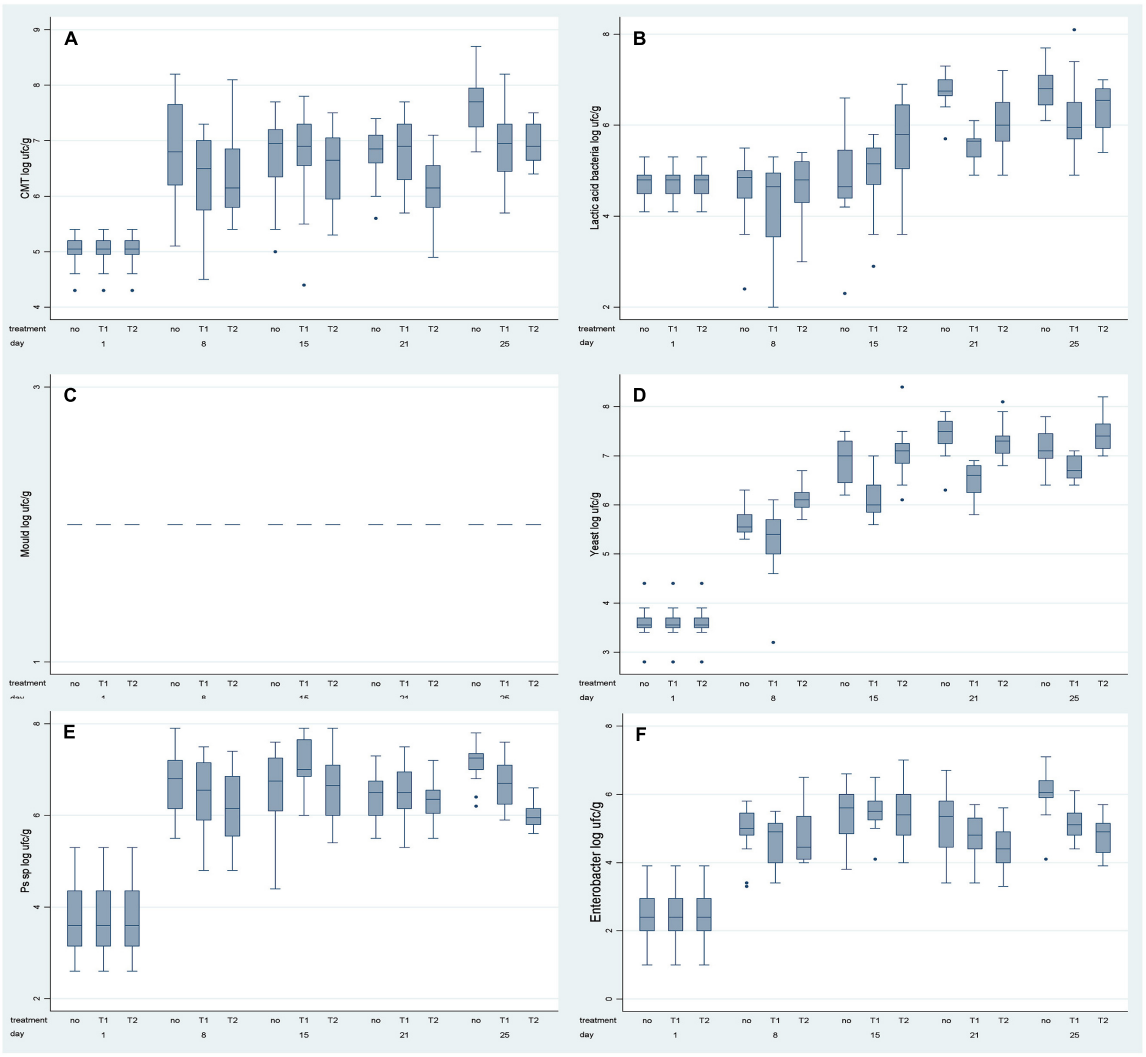
**Box plot of the microbiological data per observation time and type of packaging: traditional (no), *Ovtene*^®^ packaging (T1) and *Food-Touch*^®^ system (T2). (A)** Total Microbial Counts; **(B)** Lactic Acid Bacteria; **(C)** Moulds; **(D)** Yeasts; **(E)** *Pseudomonas* sp.; **(F)** *Enterobacteriaceae*.

The population dynamics of spoilage-related microorganisms (Total Viable Counts, lactic acid bacteria, moulds, yeasts, and *Pseudomonas* spp.) and *Enterobacteriaceae* in the *Stracchino* cheese are described in **Figure 1**.

The assumptions of homoscedasticity of data and the normality of residuals were satisfied for each analyzed microbial group (data not shown).

Analysis of variance analysis showed that populations of each of the investigated groups of microorganisms were affected both by storage time (as expected) and by type of packaging (*P* < 0.001).

In particular, Total Viable Counts increased initially (between 0 and 15 days of storage), maintained a constant value during days 15–21, and then increased further during days 21–25 of storage (**Figure 1A**). In detail, in traditionally packaged cheese, the Total Viable Counts were significantly higher than in *Ovtene*^®^-packaged cheese on days 8 and 25. Total Viable Counts in cheese packaged in the *Food-Touch*^®^ system were slightly, but significantly, lower on each sampling day than in traditionally packaged cheese. Total Viable Counts in cheese packaged in the *Food-Touch*^®^ system were slightly but significantly lower than in cheese packaged in the *Ovtene*^®^ system on each given day, except for days 8 and 25, when the cheese in the two packaging systems contained similar numbers of Total Viable Count bacteria.

Lactic acid bacteria showed a similar growth trend in cheese in the examined packaging systems (**Figure 1B**). Lactic acid bacteria numbers in cheese remained constant until day 8, and increased from day 15 until the end of storage. This trend was more evident in the case of the traditional packaging. At the end of storage, lactic acid bacteria numbers in cheese in *Ovtene*^®^ packaging were lower than in traditionally packaged cheese and in *Food-Touch*^®^-packaged cheese.

No moulds were detected in *Stracchino* cheese during the study, so these data are not discussed further.

*Ovtene*^®^-packaged cheese had noticeably lower yeast counts than cheese in traditional packaging and *Food-Touch*^®^-packaged cheese (**Figure 1D**).

*Pseudomonas* spp. increased in numbers in the cheese in all three packaging systems in the first days of storage (up to around day 8), and remained constant thereafter, except for slight fluctuations (**Figure 1E**).

Finally in the case of *Enterobacteriaceae*, a marked increase in numbers was measured in the first 15 days of storage, then slight fluctuations were observed at the end of the storage time (**Figure 1F**). *Enterobacteriaceae* numbers were lower in cheese packaged in the *Ovtene*^®^ system than in the traditional system after 25 days of storage. *Enterobacteriaceae* numbers were lower in *Food-Touch*^®^-packaged cheese between days 21 and 25 than in cheese packed in the other two systems.

### Chemical Analysis

**Table 2** shows the results of chemical analyses on cheese during storage. The only difference between the three examined packaging systems was the level of detected TVB-N. When the *Stracchino* cheese was wrapped with either one of the two active packaging options, higher levels of TVB-N were detected, compared with the traditional system. No differences were noticed for all the other investigated parameters.

**Table 2:** Chemical determinations obtained from analyses of cheese packaged in traditional, innovative and active packaging.

Experimental Time (days)	TVB-N (mgN/100 g)	Sulfides	Peroxides (MEQ O_2_/Kg of fat)	Stamm test	TBA test
**Traditional packaging**					
0	2.00 ± 1.02	Negative	<5	Negative	Negative
7	3.00 ± 1.27	Negative	<5	Negative	Negative
14	4.30 ± 6.30	Negative	<5	Negative	Negative
20	0.10 ± 0.31	Negative	<5	Negative	Negative
24	13.00 ± 8.97	Negative	<5	Negative	Negative
***Ovtene***^®^ **packaging**					
0	2.00 ± 1.02	Negative	<5	Negative	Negative
7	3.00 ± 1.06	Negative	<5	Negative	Negative
14	15.70 ± 11.26	Negative	<5	Negative	Negative
20	14.05 ± 6.51	Negative	<5	Negative	Negative
24	1.65 ± 1.42	Negative	<5	Negative	Negative
***Food-Touch***^®^ **packaging**					
0	2.00 ± 1.02	Negative	<5	Negative	Negative
7	1.00 ± 1.15	Negative	<5	Negative	Negative
14	14.80 ± 8.54	Negative	<5	Negative	Negative
20	<0.10	Negative	<5	Negative	Negative
24	2.55 ± 1.93	Negative	<5	Negative	Negative

*Mean and standard deviation for each observation time are indicated.*

### Sensory Evaluation

No significant difference due to the packaging characteristics, was displayed by the sensory evaluation tests for the identified parameters except for homogeneity and adherence, with *Ovtene*^®^ packaging displaying the best performance (*P* > 0.05; data not shown).

### Silver Migration Test

The extent of Ag migration from the *Food-touch*^®^ composite film into the cheese over time is shown in **Table 3**. The migration of Ag increased gradually from 0.053 mg/kg after 7 days of incubation to 0.103 mg/kg after 24 days (**Table 3**).

**Table 3:** Assessment of silver migration to cheese from traditional and active packaging.

Time (days)	Silver (mg/kg)
**Traditional packaging**	
0	<0.0015
7	<0.0015
14	<0.0015
20	<0.0015
24	<0.0015
***Food-Touch***^®^ **packaging**	
0	<0.0015
7	0.053 ± 0.025
14	0.245 ± 0.053
20	0.047 ± 0.023
24	0.103 ± 0.038

*Mean and standard deviation for each observation time are indicated.*

## Discussion

In this work, the effectiveness of two new active packaging systems on microbial, chemical, and sensorial qualities of *Stracchino* cheese was evaluated. Moreover, the possibility that the cheese could contain chemicals deriving from the active food packaging systems (*Ovtene*^®^ and *Food-Touch*^®^) was assessed.

Despite the Food-Touch^®^ system resulting in lower bacterial growth at some given times throughout the cheese storage, the final results did not show any significant difference in the cheese microbiota examined, of any packaged *Stracchino* cheese samples, excluding that the investigated packaging systems exerted a different inhibitory effect on the growth of spoilage microorganisms.

On the contrary, a putative effect exerted by the *Ovtene* system, which maintained two of the examined sensory characteristics, homogeneity and adherence, was observed. This effect may have been a consequence of the preservation of the functional cheese microbiota, known to be involved in the typical organoleptic properties of cheeses (Hanniffy et al., 2009; Sgarbi et al., 2013).

These results are coherent with previously published research (Incoronato et al., 2010, 2011; Morsy et al., 2014), suggesting that although application of silver based antimicrobial systems in the food industry is a widespread phenomenon, appraisal of the full potential of silver as an antimicrobial and its possible implementation in food packaging technologies is still a challenging task (Berton et al., 2014; Losasso et al., 2014).

However, since health and safety properties of many food contact materials are not fully understood, food safety should be the main concern when formulating materials for food packaging applications. Thus, according to the European Regulation (EC) No (1935/2004), efforts have to be devoted to investigate the overall migration of compounds from new packaging materials to the food, in order to elucidate the risks to humans consuming such packaged foods (de Kruijf et al., 2002; Chaudhry et al., 2008).

In this context, our results pose some safety concerns, as the level of silver migration from the active packaging system containing silver greatly exceeded the maximum established level for the migration of a non-authorised substance through a functional barrier (Commission of the European Communities, 2009).

Despite the relevance of the topic, to date, only a limited number of reports have studied the potential for silver migrating from plastic food containers, with most reports being focused on silver nanoparticles (Echegoyen and Nerín, 2013; von Goetz et al., 2013; Artiaga et al., 2015). In these studies, food containers were exposed to a number of food-simulating solutions (not real foods) under a variety of experimental conditions in an attempt to determine the possible risks for human health. Conversely, our data investigated silver migration using a real food matrix as the acceptor, and clearly showed that silver levels in cheese reached unacceptable levels, up to around 250 times higher than the 0.001 mg/kg level prescribed by EU regulation.

Even though the published reports have revealed that silver has a low tendency to migrate from the investigated materials into solutions which mimic food, under regular use conditions, several discrepancies were found in these studies, particularly with regard both to the obtained results and to the analytical methodologies used. However, unambiguous methodologies to detect and quantify the chemicals migrated from packaging are currently lacking, making it difficult for us to produce an overall assessment of results published to date.

As far as the *Ovtene* system is concerned, although this product did not display any effect in reducing the proliferation of all spoilage microorganisms, the preservation of some typical features of the cheese and the absence of any measured chemical migration into cheese could make this product interesting to the food industry.

## Conclusion

The development of innovative and active packaging systems could provide important instruments to overcome existing challenges that are associated with packaging materials, positively affecting the shelf life and the quality of foods, which will ultimately benefit both the producers and consumers. However, more in-depth research is needed in order to characterize their potential impacts on consumer health and the environment.

## Conflict of Interest Statement

The authors declare that the research was conducted in the absence of any commercial or financial relationships that could be construed as a potential conflict of interest.
