# Supplementary material for: Food safety concerns deriving from the use of silver based food packaging materials
Source: Front Microbiol. 2015 Oct 9;6:1109. doi: 10.3389/fmicb.2015.01109 (PMC4598577; doi:10.3389/fmicb.2015.01109)
Supplement: Supplementary file 1 [file Table_1.DOCX]

Table 1S. Instrumental parameters for GFAAS determination

| Parameter | Value |
| --- | --- |
| Wavelength (nm) | 328.1 |
| Slit (nm) | 0.5 |
| Measurement time (sec) | 3.0 |
| Background correction | D_2_ |
| Atomisation (t° C) | 1500 |
